# EBMonFHIR-based tools and initiatives to support clinical research

**DOI:** 10.1093/jamia/ocac193

**Published:** 2022-10-13

**Authors:** Brian S Alper

**Affiliations:** Computable Publishing LLC, Ipswich, Massachusetts, USA; Scientific Knowledge Accelerator Foundation, Ipswich, Massachusetts, USA

Dear Editors,

A 2022 scoping review provided an excellent service by identifying 203 HL7^®^ FHIR^®^-based tools and initiatives to support clinical research^1^ but did not report several developments which have not been published extensively in the peer-reviewed literature.

An HL7 project started in 2018, Fast Healthcare Interoperability Resources for Evidence-Based Medicine Knowledge Assets (EBMonFHIR), has the goal to provide interoperability (standards for data exchange) for those producing, analyzing, synthesizing, disseminating, and implementing clinical research (evidence) and recommendations for clinical care (clinical practice guidelines).^2^ FHIR Resources developed or advanced by the EBMonFHIR project that are specifically useful for supporting clinical research include Citation (for the published artifacts), ResearchStudy (for the study record), Evidence (for the study results), EvidenceVariable (for the specification of exposures and outcomes), and ArtifactAssessment (for the comments, ratings, and classifications of knowledge artifacts). These research-supporting resources are available in the Current Development build version of FHIR (FHIR Release #5 at http://build.fhir.org/).

The COVID-19 Knowledge Accelerator (COKA) Initiative is an open virtual community with 12 active working groups meeting weekly contributing to the development of standards (particularly FHIR) and tools for the computable expression of evidence and guidance.^3^^,^^4^ EBMonFHIR and COKA projects are freely available on the Fast Evidence Interoperability Resources (FEvIR) Platform at https://fevir.net which is a developing platform to support the creation, viewing, notification, and interaction for computable expression of scientific knowledge, primarily based on the FHIR current build.

Free-to-use tools to support automated conversion of structured data reporting clinical research into FHIR Resources include Computable Publishing^®^: MEDLINE-to-FEvIR Converter at https://fevir.net/medlineconvert, Computable Publishing^®^: ClinicalTrials.gov-to-FEvIR Converter at https://fevir.net/ctgovconvert, and Computable Publishing^®^: RIS-to-FEvIR Converter at https://fevir.net/ris.

A substantial vocabulary and ontology, the Scientific Evidence Code System (SEVCO), is in development at https://fevir.net/resources/Project/27845. SEVCO is an open project with a global participation and, as of July 22, 2022 has reached unanimous approval of 69 of 70 terms for study design, 121 of 260 terms for risk of bias, and 60 of 235 terms for statistics.

An important development for the support of clinical research is development of a standard for the expression of eligibility criteria for research studies. A standard for structured eligibility criteria can facilitate clinical trial matching services used or trial recruitment or to find available studies for a selected patient. Efforts with the HL7 Biomedical Research and Regulation Work Group and an EBMonFHIR Track in an HL7 FHIR Connectathon have resulted in substantial refinement of the EvidenceVariable Resource StructureDefinition (http://build.fhir.org/evidencevariable.html) and demonstrations of “Eligibility Criteria specification with EvidenceVariable” can be found at https://fevir.net/resources/Project/32444.

One week after the scoping review was published, another systematic review reported on 49 studies investigating the use of FHIR in health research.^5^ This is a rapidly developing and important area. We started a project page for “HL7 FHIR-based tools and initiatives to support clinical research” at https://fevir.net/resources/Project/52228 and encourage the scoping review authors or other interested collaborators to maintain a living review of tools and initiatives.

## AUTHOR CONTRIBUTIONS

BSA contributed the conceptualization and writing of this correspondence.
